# Prevalence and determinants of chronic kidney disease among HIV/AIDS patients in selected governmental hospitals at Addis Ababa, Ethiopia: a retrospective cross-sectional study

**DOI:** 10.1186/s12879-025-12209-2

**Published:** 2025-11-21

**Authors:** Getasew Kassaw Alemu, Nebiyu Getachew, Selam Bogale Gissa, Zemichael Getu Alemayehu, Mikiyas Gifawosen Teferi, Fitsum Assefa Gemechu, Abebe Worku Teshager, Anteneh Eshetu Berga, Bewuketu Terefe, Addisu Melkie Ejigu

**Affiliations:** 1 Nephrology Unit, Department of Internal Medicine, College of Health Sciences, Addis Ababa University, Addis Ababa, Ethiopia; 2 Department of Internal Medicine, College of Medicine and Health Science, University of Gondar, Gondar, Ethiopia; 3 Infectious Disease Unit and Yekatit – 12 Hospital Medical College, Department of Internal Medicine, College of Health Sciences, Addis Ababa University, Addis Ababa, Ethiopia; 4 College of Health Sciences, Addis Ababa University, Addis Ababa, Ethiopia; 5 Infectious Disease Unit, Department of Internal Medicine, College of Health Sciences, Addis Ababa University, Addis Ababa, Ethiopia; 6 Community Health Nursing Department, School of Nursing, College of Medicine and Health Science, University of Gondar, Gondar, Ethiopia

**Keywords:** Chronic kidney disease and HIV, Kidney dysfunction and HIV, HIV/AIDS, Ethiopia

## Abstract

**Background:**

The burden of noncommunicable diseases, particularly kidney dysfunction, is increasingly common among HIV/AIDS patients, especially with the improved life expectancy resulting from the advent of antiretroviral therapy. However, there are limited data on the prevalence and determinants of chronic kidney disease in this population. Therefore, this study aimed to assess the prevalence and determinants of chronic kidney disease among HIV/AIDS patients at two government hospitals in Ethiopia in 2024.

**Methods:**

A retrospective cross-sectional study was conducted among 422 adult HIV/AIDS patients, selected via systematic random sampling techniques with a sampling frame, at two governmental hospitals at Addis Ababa, Ethiopia. The data were collected via the kobo tool box software, and the analysis was performed via Stata software version 17. Variables with a p-value of *≤* 0.2 in the bivariate analysis were included in the multivariable logistic regression model, and variables with a p-value of less than 0.05 in the final model were considered statistically significant.

**Results:**

Among the 422 participants, 54 (12.8%) were excluded for having inadequate kidney function determination and 368 participants were included in the final analysis. The prevalence of chronic kidney disease in this study was 59 (16.03%). Those individuals with higher education (AOR = 0.53, 95% CI: 0.39–0.93) and self-employed status (AOR = 0.83, 95% CI: 0.33–0.98) were less likely to have chronic kidney disease. Age > 64 years was (AOR = 2.99, 95% CI: 1.05–8.57), antiretroviral therapy duration > 20 years was (AOR = 5.84, 95% CI: 3.77–14.53), being widowed was (AOR = 1.32, 95% CI: 1.04–3.88), and diarrheal disease in the study period of (AOR = 1.27, 95% CI: 1.06–3.27) times was significantly associated with the occurrence of chronic kidney disease.

**Conclusions:**

The prevalence of chronic kidney disease in this study was relatively high compared to national and regional estimates. Significant factors associated with chronic kidney disease included older age, longer duration on antiretroviral therapy, history of diarrheal disease, being widowed, higher educational status, and self-employment. These findings highlight the need for the integration of routine kidney function screening into HIV care services to support early detection and management of chronic kidney disease in this population.

**Clinical trial number:**

Not applicable.

## Introduction

Human immunodeficiency virus (HIV) infection had significantly affected the world causing over 40 million deaths since the 1980s and reshaping the global public health priority and over 38 million people had the infection currently [1]. This had caused significant challenges by affecting the working-age group, causing considerable impact on the socioeconomic structure of families and the community at large, and challenging the healthcare system; the devastating impact was noted in Sub-Saharan Africa, where the majority of acquired immunodeficiency syndrome (AIDS)-related morbidity and mortality cases had occurred globally [2, 3]. Unwavering international cooperation’s from the Presidents Emergency Plan for AIDS Relief (PEPFAR), United Nations Program on HIV/AIDS (UNAIDS), the global fund and others had led the major advances in the accessibility of HIV/AIDS treatments in Africa; saved millions of lives [4, 5].

However, advancements in antiretroviral therapy (ART) had improved life expectancy accompanied by the emergence of noncommunicable disease (NCD) including chronic kidney disease (CKD) [6–9]. Kidney dysfunction was observed in approximately 30% of HIV-infected people at some stage of their disease, making it a relatively common indication for dialysis [10, 11]. People living with HIV (PLWH) were 2-20-fold more likely to be at risk of kidney disease than the general population [12, 13]. The prevalence of CKD conducted among HIV infected individuals in the United States of America (USA) was 3% [14], that in Brazil 8.4% [15], that in China 3.3% [16] and 5.8% [17], that in Italy 13.3% [18] and 21.3% [19], that in Nigeria 15.3% [20], that in Uganda 2.5% [11], that in Cameroon 19.1% [21], and that in Tanzania 7% [22]. Four studies in Ethiopia had reported inconsistent results; 22.1% from Mehal Meda Hospital [23], 20.7% from Metu Hospital [24], and 16.1% from Gondar Hospital [25], and the oldest study conducted at Addis Ababa University revealed no relationship between HIV infection and kidney function impairment [26].

The findings of these studies involved one-time kidney function determinations, especially those studies conducted in Ethiopia.

HIV-infected patients could develop different forms of renal disease, including acute kidney injury (AKI), CKD, glomerular disease, urologic disorders or interstitial inflammation [10, 27]. HIV-associated nephropathy (HIVAN) was the most commonly recognized kidney disease associated with HIV infection, particularly among people of African descent, due to genetic predispositions [10, 28]. The burden of CKD among HIV/AIDS patients could be due to HIV- and Non-HIV-related risk factors. Sociodemographic characteristics such as sex, age, history of kidney disease, medical comorbidities, hepatitis virus coinfections, medications or any episode of AKI of any reason were associated with the occurrence of HIV related kidney dysfunction [11, 29]. Low CD4 counts, high HIV viral counts, tenofovir (TDF) and, ART duration were also HIV-associated determinants of kidney dysfunction [11]. Therefore, considering this burden and all these risk factors, the World Health Organization (WHO) and Infectious Disease Society of America (IDSA) had recommended monitoring kidney function annually and even more frequently in patients taking TDF-based regimens [10].

The burden of renal dysfunction was increasing alongside other NCDs among HIV/AIDS patients with significant mortality and morbidity. This phenomenon was considerably high in Sub-Saharan Africa, including Ethiopia. The ART era is accompanied by an increasing number of HIV-infected patients with kidney dysfunction both globally and locally. This highlights the importance of integrating NCD care with HIV/AIDS services rather than managing patients in isolation, as such integration can offer multiple clinical and health system benefits. However, despite the high burden of impaired kidney function, the integration of kidney function monitoring into HIV/AIDS care remains limited in resource-constrained settings, as demonstrated by the few studies conducted in Ethiopia. This multicenter retrospective cross-sectional study, conducted at Tikur Anbessa Specialized Hospital (TASH) and Yekatit 12 Hospital Medical College, assessed the prevalence and determinants of CKD among HIV/AIDS patients. The findings of this study provide valuable evidence supporting the integration of kidney function determination into the routine HIV/AIDS care package. Additionally, the study assessed the current level of integration of kidney function monitoring within routine HIV/AIDS services.

## Methods and materials

### Study design, setting and period

This was a multicenter institution-based retrospective cross-sectional study conducted at Tikur Anbessa Specialized Hospital (TASH) and Yekatit 12 Hospital Medical College, Addis Ababa, Ethiopia. TASH is the largest tertiary hospital in the country, offering subspecialty-level care across a broad range of medical disciplines and serving as a major teaching hospital under Addis Ababa University. Yekatit 12 Hospital Medical College is one of the country’s oldest hospitals and continues to serve both as a healthcare provider and training institution. HIV/AIDS patients receive follow-up care across multiple centers in Addis Ababa; however, these two hospitals were purposively selected due to their high patient volumes and the availability of comprehensive HIV/AIDS services. The study period spanned from June 1st, 2022, to July 31st, 2024; during this time, patient records were retrospectively reviewed to extract data from the most recent clinical encounter within the interval. Although the review covered a two-year period, the data reflect a cross-sectional snapshot for each patient, and no follow-up or repeated measures were involved.

### Source and study populations

The source population included all adult HIV/AIDS patients (aged ≥ 18 years) who were registered and receiving chronic HIV care and follow-up at Tikur Anbessa Specialized Hospital (TASH) and Yekatit 12 Hospital Medical College during the study period. From this broader group, the study population was selected using a systematic random sampling method and consisted of patients with complete and accessible medical records between January 2022 and March 2024. Only those who met the inclusion criteria—such as having a confirmed HIV/AIDS diagnosis and being actively followed at the respective institutions—were included in the final analysis.

### Inclusion criteria

All adults (aged *≥* 18 years), diagnosed with HIV/AIDS and on follow-up at either TASH or Yekatit 12 Hospital Medical College during the study period were eligible.

### Exclusion criteria

We excluded patients with no recorded serum creatinine, those with creatinine results lacking a documented measurement date, and those with unreadable values.

## Sample size determination

### Sample size

The sample size was initially calculated using the single population proportion formula, based on the assumption of a 50% prevalence of chronic kidney disease (CKD), with a 95% confidence level (Z = 1.96) and a 5% margin of error (d = 0.05), which yields a minimum sample size of 384 participants. To account for a potential 10% non-response or data incompleteness, the final target sample size was adjusted to 422.

However, during data collection, only 368 patient records were eligible for inclusion due to incomplete or missing data in 54 charts, particularly regarding key variables required for analysis (e.g., creatinine results, ART duration, or demographic details). Extending the data collection period or replacing excluded records was not feasible due to time and resource constraints, as well as limited availability of additional eligible and complete records during the study window.

Despite the reduced sample size, 368 participants still represent over 87% of the originally calculated sample and are considered sufficient to maintain acceptable statistical power for the main analyses. Moreover, the systematic sampling method ensured that the final sample remained representative of the overall patient population attending the two facilities. The formula used is as follows: where *P* = 50%, d = 5%.

$$n{\rm{ }} = {{{{\left( {Z\alpha /2} \right)}^2}\left( P \right){\rm{ }}\left( {1 - p} \right)} \over {{d^2}}}$$

$${n_1} = {{{{\left( {1.96} \right)}^2}x{\rm{ }}0.5{\rm{ }}\left( {1 - 0.5} \right)} \over {0.0025}}\, = \,384$$

Adding 10% compensate for potential data loss/ineligibility during abstraction, the total sample size was 422.

### Sampling procedures

In this study, a systematic random sampling technique was employed to select patient records from two hospitals: Tikur Anbessa Specialized Hospital (TASH) and Yekatit-12 Hospital Medical College. The total number of HIV/AIDS patients on follow-up during the study period (January 2022 to March 2024) was approximately 7000 at TASH and 3300 at Yekatit-12, though slight variation may have occurred due to patient transfers, loss to follow-up, or new enrollments.

To ensure proportional representation, the sample size was allocated based on the patient load at each hospital—two-thirds (281 patients) from TASH and one-third (141 patients) from Yekatit-12. The sampling interval (*K*) was calculated by dividing the total number of eligible patients by the allocated sample size, resulting in a K-value of 25 at TASH and 24 at Yekatit-12. The first record in each hospital was selected using a lottery method from the first 25 or 24 patients, respectively, to ensure randomization. Subsequently, every *K*th record was selected until the desired sample size was reached.

Medical records were accessed through the ART clinics of both hospitals in collaboration with clinical staff. Data were collected retrospectively using a structured and pre-tested data extraction checklist, designed to capture key variables including socio-demographic details, clinical parameters, comorbidities, ART duration, and laboratory results (such as serum creatinine). The checklist was pre-tested on 5% of records at a comparable facility, and adjustments were made accordingly.

Trained health professionals conducted the data collection, and the process was supervised daily by the principal investigator to ensure completeness and accuracy. All incomplete or illegible records were excluded from the analysis.

### Study variables

#### Dependent variable

chronic kidney disease (CKD).

### Independent variables

#### Sociodemographic

Age, sex, address, occupation, education, marriage.

#### Comorbidities

Diabetes mellitus, hypertension, heart failure, stroke, dyslipidemia, malignancy, hepatitis B virus (HBV), hepatitis C virus (HCV), seizure disorders, and obstructive uropathy.

#### Medications

ART regimen, ART duration, use of any antibiotics, nonsteroidal anti-inflammatory drugs (NSAIDs), tuberculosis preventive therapy (TPT), cotrimoxazole prophylactic therapy (CPT),

#### HIV/AIDS related factors

HIV infection duration, initial and current WHO stage, baseline CD4 count, recent viral load, baseline body mass index, and opportunistic infection during the study period, and proteinuria during the study period, and adherence to ART.

### Operational definition

After the estimated glomerular filtration rate (eGFR) was calculated using the CKD-Epidemiology Collaboration (CKD-EPI) equation, chronic kidney disease (CKD) was defined as a sustained eGFR of < 60 ml/min/1.73 m² based on at least two creatinine measurements separated by a minimum of 3 months, in accordance with KDIGO guidelines [1]; Proteinuria was defined as >+ 1 on dipstick urinalysis [2]. Histories of opportunistic infections were considered based on documented physician diagnoses. Hypertension and diabetes mellitus were defined by the documented use of medications for those conditions. Hepatitis C virus (HCV) and hepatitis B virus (HBV) positivity were defined based on HCV antibody and HBV surface antigen test results, respectively. The nutritional status of HIV/AIDS patients is according to national consolidated guideline for comprehensive HIV prevention, care and treatment in Ethiopia guidelines description which classified as normal/appropriate, mild, moderate or severe malnutrition.

### Data collection procedures and quality assurance

Data were collected from the patients and their medical records (charts, registry reports and electronic databases) via a data abstraction tool that consists of all the above exposure and outcome variables. A pretest was performed on 5% of the randomly selected medical charts, and the necessary revision of the data collection tool was made on the basis of the test results.

Data collection was performed via the kobo toolbox software. The principal investigator had provided the training on the basics of the data abstraction form and how to use the form and software appropriately for three general practitioners. The consistency and completeness of the collected data was checked before any attempt to analyze the data.

The outcome variable was serum creatinine, and an eGFR was calculated using the CKD-EPI equation. The serum creatinine level was collected from the document after the patient interview, and the values in the year 2022–2024 (2 years) were collected. If there are multiple measurements, the lowest value was taken, and if there was no measurement at all or incomplete, it was considered as incomplete to define CKD. During the data collection process, patients were not asked to pay for the purpose of this research. The investigations, renal function specifically, was taken from the documents.

### Data management and analysis

The data were initially exported from Kobo Toolbox into Microsoft Excel, where they were manually checked, cleaned, sorted, categorized, and coded. After cleaning, the dataset was imported into STATA version 17 for statistical analysis. Descriptive statistics, including frequencies, proportions, means, and standard deviations, were used to summarize the characteristics of the study population. To identify factors associated with chronic kidney disease, both bivariate and multivariable logistic regression analyses were conducted. Variables with a p-value of *≤* 0.2 in the bivariate analysis were included in the multivariable logistic regression model, and variables with a p-value of less than 0.05 in the final model were considered statistically significant. Although the study involved multiple health centers, we assumed that both the centers and the study participants were relatively homogeneous with respect to the key clinical and demographic characteristics. Based on this assumption and the absence of observable inter-center variability, we applied a fixed-effects logistic regression model. Random-effects or multilevel models were not employed, as no clustering effects were identified. To further enhance the validity of our model and reduce potential bias, we selected variables based on prior literature, applied stratification where appropriate, and assessed multicollinearity using the Variance Inflation Factor (VIF), with all VIF values falling below the accepted threshold.

## Results

### Demographic characteristics

Among the 422 participants, 54 (12.8%) were excluded, 36 individual patients from TASH and 18 individual patients from Yekatit-12 Hospital Medical College. The mean age of the participants was 44.88 *±* 13.59 years (SD). Among the 368 enrolled participants with creatinine data, 229 (62.23%) were females, 335 (91.03%) were from Addis Ababa, 175 (47.55%) were self-employed, 148 (40.22%) were married, 32 (8.7%) were students, and more than 50% of the participants with HIV/AIDS had secondary or higher education levels. The patients were homogenous, individuals who were black, although relative cultural differences could be noted (Table 1).

**Table 1:** Demographic characteristics of participants with HIV/AIDS in Addis Ababa, Ethiopia, 2024 (*n* = 368)

Variable	Category	Observation	
		*N*	%
Hospital, center	TASH Yekatit-12 Hospital Medical College	245 123	66.6 33.4
Age	Mean age was 44.88 SD (*±* 13.59)		
Gender	Male Female	139 229	37.8 62.2
Education	Higher Secondary Primary No formal education	88 154 94 32	23.9 41.9 25.8 8.4
Occupation	Government employed Self employed Daily laborer House wife Student	48 175 58 55 32	13.0 47.6 15.8 15.0 8.7
Marriage	Married Single Divorced Widowed	148 102 53 65	40.2 27.7 14.4 17.7
Address	Addis Ababa Others*	335 33	91.0 9.0

Others* represent regions other than Addis Ababa

### Comorbidity related characteristics

Among all the participants, 308/368 (83.7%) had reported none of the medical comorbidities. Among those patients with comorbidities (16.3%), hypertension and diabetes mellitus were the most frequently reported ones. The presence of one medical comorbidity was likely associated with presence of the other comorbidity as shown in the table where multiple comorbidities were observed frequently in few of the HIV/AIDS patients. Therefore, a single HIV/AIDS patient was observed to have more than one medical comorbidity concomitantly and the list of OIs’ observed in this study are shown on the table below (Table 2).

### Opportunistic infections or stage defining disease

Among the 368 study subjects, 279 (75.8%) had reported none of the OIs’ during the study period. Among those with HIV/AIDS patients who had OI (24.2%), candidiasis, tuberculosis, diarrheal disease and pneumonia were observed frequently during the study period. Those HIV/AIDS patient with one OI were likely to have more OIs’ concomitantly and the list of OIs’ observed is shown on the table below (Table 2).

### HIV/AIDS related clinical and laboratory characteristics

The mean duration of HIV infection was 15.64 *±* 4.09years. The number of HIV/AIDS patients with current WHO class I is better than the baseline WHO class 1 (99.2% vs. 62.0%) indicating the possible efficacy of ART from good (G) adherence reported among 97.6%. Nutritional assessment showed that 292 (79.4%) of the subjects had normal/appropriate nutritional status. The baseline CD4 count was *≥* 200 in 250/368 (67.9%). Among, those small number of HIV/AIDS patients who had dipstick urinalysis (137/368), 52 (14.1%) had proteinuria (Table 2).

### Medications profile

In this study, HIV/AIDS patients were exposed to ART and non-ART medications which could affect their kidney health directly or indirectly. TDF based ART regimen was used by 307/368 (83.4%) and TDF + 3TC + DTG was the most commonly used ART regimen (76.4%). Significant number of patients were exposed to TPT and CPT. Antibiotics and NSAID were also used by many more HIV/AIDS patients. A single HIV/AIDS patient could be exposed to one or more of these medications once or at different intervals (Table 2).

**Table 2:** Baseline characteristics of HIV/AIDS patients in addis Ababa, Ethiopia, 2024 (*n* = 368)

Variable	Category	Observation		
		Frequency	%	
Comorbidity	No medical comorbidity	308	83.7	
	Hypertension + Diabetes mellitus	12	3.2	
	Hypertension + Diabetes mellitus + dyslipidemia	9	2.4	
	Hypertension + Diabetes mellitus + heart failure	9	2.4	
	Hypertension + Diabetes mellitus + Asthma/COPD	10	2.7	
	Hypertension + HBV + obstructive uropathy	3	0.8	
	HCV + obstructive uropathy	3	0.8	
	Hypertension + malignancy	4	1.1	
	Hypertension + stroke + Seizure disorder	6	1.6	
	Seizure disorder + obstructive uropathy	4	1.1	
HIV/AIDS related (Clinical)	Baseline HIV/AIDS WHO class	I II III IV	228 40 76 24	62.0 10.9 20.6 6.5
	Current WHO class	I II	365 3	99.2 0.8
	ART duration (years), Mean = 14.63 *±* SD (= 4.01)	1–9 10–20 > 20	51 311 6	13.9 84.5 1.6
	ART adherence	Good (G) Fair (F) Poor (P)	359 5 4	97.6 1.4 1.0
	HIV infection duration (years) Mean = 15.64 *±* SD (4.09)	1–9 10–20 > 20	39 305 24	10.6 82.9 6.5
	Body mass index (BMI)	Normal Mild malnutrition Moderate malnutrition Severe malnutrition	292 36 30 10	79.4 9.8 8.2 2.6
HIV-related (Laboratory)	Baseline Hgb < 13 Baseline Hgb *≥* 13	No Yes	343 25	93.2 6.8
	Baseline CD4	>=200 < 200	250 118	67.9 32.1
	Current viral load	Undetected Detectable Unknown	160 190 18	43.5 51.6 4.9
	Proteinuria (urine dipstick)	Negative >+1 Not done	85 52 231	23.1 14.1 62.8
Patients with opportunistic Infection (OI))		None Yes	279 89	75.8 24.2
Frequently reported OIs More than one OI could be reported by a single patient.		Tb + PCP + Pneumonia + Diarrheal disease + Candidiasis	43	11.7
		Toxoplasmosis + Meningitis + Diarrheal disease	9	2.4
		Herpes zoster + Candidiasis	14	3.8
		Candidiasis only	9	2.4
		Cervical cancer + Lymphoma + Candidiasis	14	3.8
Non-ART Medications Profile in the study period	No medication intake than ART reported		59	16.0
	Tb preventive therapy (TPT)		78	21.2
	Cotrimoxazole preventive therapy (CPT)		74	20.0
	Nonsteroidal anti-inflammatory drugs (NSAID)		78	21.2
	Antibiotics		79	21.5
TDF use	Yes No		307 61	83.4 16.6
ART regimen used during the study period		TDF + 3TC + DTG TDF + 3TC + ATV/r AZT + 3TC + ATV/r ABC + 3TC + DTG TDF + 3TC + EFV AZT + 3TC + DTG Others *	281 17 16 10 9 7 28	76.4 4.6 4.4 2.7 2.4 1.9 7.6

Abbreviation and symbols: ABC -Abacavir, AIDS-acquired immune deficiency syndrome, ART- anti retroviral therapy, ATV/r-Atazanavir, AZT-zidovudine, CD4-cluster of differentitiation4, COPD-chronic obstructive pulmonary disease, DTG-dolutegravir, EFV-efavirenz, HBV-hepatitis B virus, HCV-hepatitis C virus, HGB-hemoglobin, HIV-human immunodeficiency virus, PCP – pneumocystis carinii Pneumonia, SD-standard deviation, Tb – tuberculosis, 3TC-lamuvidine, WHO- world health organization. The symbol + in the table represents sum or plus or addition of the variables there

### Prevalence of chronic kidney disease (CKD)

**Fig. 1:**
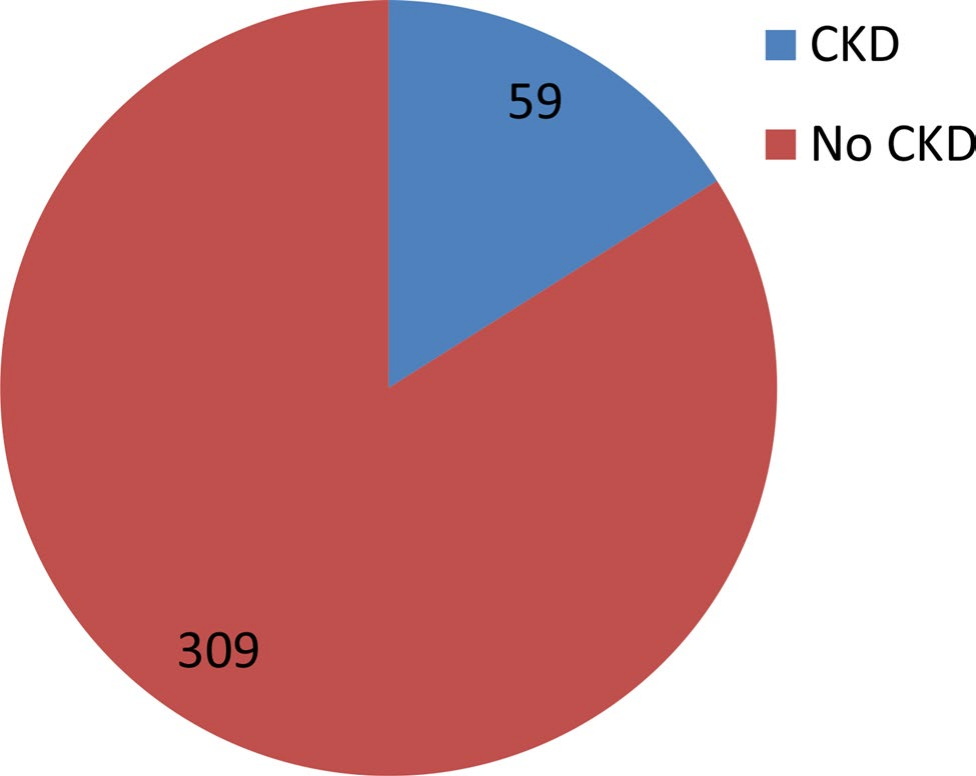
Prevalence of CKD among HIV/AIDS patients in Addis Ababa, Ethiopia, 2024 (*N* = 368)

The prevalence of chronic kidney disease among HIV/AIDS patients was 59 (16.03%) of which 44 (74.6%), 11 (18.6%) and 4 (6.8%) were in G3, G4 and G5 category respectively. Among the patients with chronic kidney disease, 37 (62.71%) were females, and 22 (37.29%) were males (Table 3; Fig. 1).

**Table 3:** Distribution of HIV/AIDS patients in relation to the outcome in Addis Ababa, Ethiopia, 2024 (*n* = 368)

Characteristics	Variable	Category	CKD (*n* = 59)	No CKD (*n* = 309)
Hospital	TASH Yekatit- 12 medical college		37 22	197 101
Demographic	Age	< 50	27	197
		50–64	23	95
		> 64	9	17
	Sex	Female	37	192
		Male	22	117

### Factors associated with CKD among HIV patients

Binary and multivariable logistic regression analysis revealed several significant variables associated with CKD burden among HIV/AIDS patients in Addis Ababa, Ethiopia, with 95% confidence intervals. Those individuals with higher education (AOR = 0.53, 95% CI: 0.39–0.93) and self-employed status (AOR = 0.83, 95% CI: 0.33–0.98) were less likely to have chronic kidney disease. Age > 64 years was (AOR = 2.99, 95% CI: 1.05–8.57), ART duration > 20 years was (AOR = 5.84, 95% CI: 3.77–14.53), being widowed was (AOR = 1.32, 95% CI: 1.04–3.88), and diarrheal disease in the study period of (AOR = 1.27, 95% CI: 1.06–3.27) times was significantly associated with the occurrence of CKD (Table 4).

**Table 4:** Univariate and multivariable logistic regression analyses on factors associated with prevalence of CKD among HIV/AIDS patients in addis Ababa, Ethiopia, 2024 (*n* = 368)

Variable	COR (95% CI)	AOR	95% CI for AOR	*P*-value
**Education**				
Higher	1.14 (0.41, 3.19)	0.53	(0.39, 0.93)	0.027*
Secondary	0.81 (0.31, 2.17)	0.87	(0.44, 2.53)	0.775
Primary	0.44 (0.14, 1.34)	0.51	(0.15, 1.74)	0.287
Not educated (ref)	1	1		
**Occupation**				
Unemployed (ref)	1	1		
Employed	1.43 (0.56, 3.59)	1.17	(0.40, 3.41)	0.773
Housewife	0.85 (0.32, 2.21)	1.46	(0.47, 4.47)	0.514
Self-employed	0.68 (0.31, 1.48)	0.83	(0.33, 0.98)	0.042*
Student	0.44 (0.11, 1.72)	0.57	(0.12, 2.71)	0.482
**Age**				
< 50 (ref)	1	1		
50–64	1.77 (0.96, 3.24)	1.37	(0.68, 2.74)	0.375
> 64	3.86 (1.57, 9.52)	2.99	(1.05, 8.57)	0.041*
**ART Duration (years)**				
< 10 (ref)	1	1		
10–20	1.81 (0.68, 4.76)	1.45	(0.51, 4.11)	0.486
> 20	9.20 (1.45, 28.35)	5.84	(3.77, 14.53)	0.002*
**CD4 Count**				
< 200 (ref)	1			
> 200	1.32 (0.71, 2.46)	1.33	(0.66, 2.65)	0.422
**Proteinuria**				
No (ref)	1			
Yes	0.58 (0.28, 1.19)	0.83	(0.36, 1.91)	0.661
**Marital Status**				
Single (ref)	1	1		
Married	0.89 (0.38, 2.09)	0.86	(0.34, 2.21)	0.752
Divorced	0.71 (0.28, 1.79)	0.92	(0.31, 2.76)	0.885
Widowed	1.34 (0.53, 3.39)	1.32	(1.04, 3.88)	0.006*
**Baseline BMI (kg/m²)**				
Normal (ref)	1	1		
Malnutrition	1.79 (0.81, 3.97)	0.54	(0.23, 1.28)	0.161
**Heart Failure**				
No (ref)	1	1		
Yes	2.71 (0.66, 11.13)	1.85	(0.33, 10.41)	0.482
**Malignancy**				
No (ref)	1	1		
Yes	5.39 (0.74, 39.01)	5.16	(0.57, 45.90)	0.145
**Diarrheal Disease**				
No (ref)	1	1		
Yes	0.27 (0.06, 1.17)	1.27	(1.06, 3.27)	0.036*
**Isoniazid Preventive Therapy**				
No (ref)	1	1		
Yes	1.34 (0.69, 2.62)	1.40	(0.65, 2.98)	0.390

Abbreviation and symbols: COR = Crude Odds Ration, AOR = Adjusted Odds Ratio, ART – antiretroviral therapy, BMI – Body mass index, CD4 – Cluster of differentiation 4; ref - reference. * represent significantly associated variables

## Discussio

The prevalence of CKD among HIV/AIDS patients was 16.03%. This finding must consider the 54 (12.8%) patients with HIV/AIDS who never had even one kidney function determination in the study period while more than 93% of patients were on TDF-based regimen, which are expected to have more frequent determination of kidney function. The burden of CKD might differ if the determination had been performed according to the WHO or IDSA recommendation [3, 4]; this kind of overlooking the kidney function was noted in the Zambian study [5] and may be a common ground fact in many low-income countries. The findings of this study (16.03%) were consistent with those of studies conducted in Nigeria (15.3%) and Ethiopia (16.1%) [6, 7]. However, the result of this study revealed a greater prevalence than did studies in the USA (3%), Brazil (8.4%), and China (3.3%) [8–10], and the prevalence was lower than the finding in the study conducted at Italy (21.3%), Cameroon (19.1%), and Ethiopia (20.7% and 22.1%) [11–13]. The discordant findings in these studies may be due to the difference in patient population, and access to healthcare. The definition of CKD and the method of data collection used in each study also differ, which significantly affects the prevalence of CKD. However, the finding in this study is much higher than the finding from a systematic review and a meta-analysis from 60 countries involving 209,078 HIV patients where the overall CKD prevalence was 6.4% [14].

In this study, the multivariable logistic regression analysis had identified several significant variables associated with CKD burden among HIV/AIDS patients using a 95% confidence interval. The presence of diarrheal disease, being widowed, aged > 64 years and being on ART for more than 20 years were associated with the occurrence of CKD, while individuals with self-employed income status and higher education levels had a lower probability of having of CKD.

Higher education (AOR = 0.53, 95% CI: 0.39–0.93) was one of the significant factors shown to be associated with less probability to develop CKD highlighting the role of education on health care and prevention of CKD. This inverse relationship between education and CKD risk could be partly explained the impact of education on healthy lifestyle and treatment adherence. The finding in this study has been also clearly implicated on a longitudinal study among 861 participants [15]. The impact of higher education to reduce vascular disease risk and vice versa had also been clearly noted in other studies [16]. Those individuals with a better educational opportunity are likely to have less risk of CKD implicating that a better awareness reduces vulnerability of individuals to CKD.

Age above 64 (AOR = 2.99, 95% CI: 1.05–8.57) had also played a significant role by increasing the probability of CKD risk among this population, highlighting age as a major risk factor for CKD in this population. The association between older age and occurrence of CKD had been implicated in many studies [8, 10, 11, 13, 17, 18] and the odds of developing CKD is low among individuals with age below 40 [19]. More importantly, the prevalence of CKD increases along with age of the individual though the cut of age varies from study to study probably associated with higher metabolic and vascular risks [20]. Firstly, aging is associated with progressive decline in renal function due to nephron loss, glomerulosclerosis, and reduced renal blood flow, which together decrease the kidney’s ability to filter blood effectively. Secondly, older individuals have a higher burden of comorbidities such as hypertension, diabetes, and cardiovascular disease, which are key contributors to CKD development. Thirdly, HIV-infected older adults may experience cumulative exposure to nephrotoxic ART drugs, chronic inflammation, and immune activation, further exacerbating kidney damage. Additionally, vascular stiffening and endothelial dysfunction common in elderly patients impair renal perfusion and contribute to kidney injury. Therefore, the increased CKD risk in those over 64 is multifactorial, involving age-related structural kidney changes, higher prevalence of metabolic and vascular comorbidities, and HIV-specific factors, underscoring the need for close renal monitoring and tailored management in this vulnerable group.

Similarly, the findings in this study had demonstrated an independent strong association between duration of ART more than 20 years (AOR = 5.84, 95% CI: 3.77–14.53) and risk of CKD. A relatively recent study had shown that a median duration of 4.5 years on ART was associated with risk of CKD in this population [21] and the other study had shown a median duration of 4.8years [22]. The risk of CKD among HIV/AIDS patients usually increase after 6 months of ART associated with TDF related nephrotoxicity [23] while other studies had shown that kidney dysfunction is dependent on the cumulative dose of TDF [24, 25].

HIV/AIDS patients who had diarrheal disease during the study period were found to be significantly associated with an increased risk of CKD (AOR: 1.27, 95% CI: 1.06–3.27). HIV/AIDS patients can have diarrheal disease frequently. Diarrhea is a common cause of pre renal AKI and may be a cause for acute deterioration in already existing CKD. One of the long-term consequences of AKI is CKD, especially if the hypovolemia is not corrected timely [26, 27].

Finally, this study had shown that self-employed (AOR = 0.83, 95% CI: 0.33–0.98) had lower probability of CKD while widowed (AOR: 1.32, 95% CI: 1.04–3.88) individuals had a greater likelihood of having CKD. The role of occupational level to reduce the risk of CKD had been identified [28]. The impact of marital status and its psychological consequence including depression is common and therefore CKD risk alongside with it [29].

This possibly shows the association between risks of CKD among those economically, socially and psychologically unsupported individuals. The impact of economic self-sufficiency and social support on health-related quality of life, which directly affects the health of individuals with HIV/AIDS has been documented [30, 31].

### Strength and limitation

This research highlighted the prevalence and determinants of CKD among HIV/AIDS patients. Importantly, the results also revealed that a significant number of HIV/AIDS patients had no kidney function determination per the WHO or IDSA recommendations. Although, this was a multicenter study, it was a retrospective study in which data incompleteness could be a major challenge. Additionally, creatinine determination was not from similar laboratory lacking standardization and uniformity for interpretation; it would have been better if the assay was similar from the same laboratory. This study also did not address the cofounders for the presence of CKD including diabetes mellitus and hypertension or other comorbidities.

## Conclusion

The prevalence of chronic kidney disease was found to be moderately high even as compared to the overall CKD burden globally. Participants with elderly age, longer ART duration, diarrheal diseases and being widowed were associated significantly with the occurrence of CKD among this populations while those with higher educational status and self-employment economic status were associated significantly with less risk of CKD. In addition to creating awareness for healthcare providers about the risk of CKD in HIV/AIDS patients, integration of regular kidney function determination per the WHO recommendation is better practiced to detect CKD early in this population; hence policy makers are need to consider this test to be included in the HIV/AIDS package of care. To obtain a better evidence, future researchers may do further research using better study designs.

## Data Availability

The data supporting this study can be requested from the corresponding author upon reasonable request.

## Abbreviation

AIDS: Acquired immunodeficiency syndrome
AKI: Acute kidney injury
ART: Antiretroviral therapy
BMI: Body mass index
CD4: Cluster of differentiation 4
CKD: Chronic kidney disease
COPD: Chronic obstructive airway disease
CPT: Cotrimoxazole preventive therapy
DTG: Dolutegravir
ESKD: End stage kidney disease
GFR: Glomerular filtration rate
HBV: Hepatitis B virus
HBsAg: Hepatitis B virus surface antigen
HCV: Hepatitis C virus
HIV: Human immunodeficiency virus
HIVAN: HIV associated nephropathy
IDSA: Infectious disease society of America
IRB: Institutional review board
NCD: Non communicable disease
NSAID: Nonsteroidal anti-inflammatory drug
OI: Opportunistic Infection
PEPFAR: Presidents emergency plan for AIDS relief
PLWA: People living with HIV/AIDS
PJP: Pneumocystis jirovecii pneumonia
SD: Standard deviation
TASH: Tikur anbesa specialized hospital
TDF: Tenofovir disoproxil fumarate
TPT: Tuberculosis preventive therapy
UNAIDS: United Nations program on HIV/AIDS
WHO: World Health Organization
